# Smart bioactive hydrogels for myocardial infarction repair: a multifunctional approach integrating stimuli-responsive drug delivery, electroconductivity, and real-time biosensing

**DOI:** 10.1097/MS9.0000000000004284

**Published:** 2025-11-18

**Authors:** Abubakar Nazir, Awais Nazir, Naseer Khan, Muhammad Shah Wali Jamal, Abdalhakim Shubietah, Imran Naqvi, Syed Rafay Hussain Zaidi, Behram Khan, Ghulam Mujtaba Ghumman, Ahmed Jamal Chaudhary, Syed Sohail Ali

**Affiliations:** a Department of Medicine, King Edward Medical University, Pakistan; b Department of Medicine, The Jewish Hospital of Cincinnati, Cincinnati, OH, USA; c Oli Health Magazine Organization, Research, and Education, Kigali, Rwanda; d Department of Medicine, University of Cincinnati, Cincinnati, OH, USA; e Department of Medicine, Advocate Illinois Masonic Medical Center, Chicago, IL, USA; f Department of Internal Medicine, UCHealth Parkview Medical Center, Chicago, CO, USA; g Department of Internal Medicine, Rochester Regional Health, Rochester, NY, USA; h Department of Internal Medicine, Mercy Health Saint Vincent Medical Center, Toledo, OH, USA; i DMC Sinai-Grace Hospital, Detroit, MI, USA

**Keywords:** 3D/4D printing, cardiac tissue engineering, electroconductive hydrogels, myocardial infarction repair, smart bioactive hydrogels, stimuli-responsive drug delivery

## Abstract

**Background:**

Myocardial infarction (MI) remains a leading cause of morbidity and mortality worldwide, necessitating advanced therapeutic strategies for cardiac repair. Conventional treatments often fail to restore cardiac function effectively, highlighting the need for innovative biomaterials. Smart bioactive hydrogels have emerged as promising candidates due to their ability to provide structural support, controlled drug delivery, electroconductivity, and real-time biosensing capabilities.

**Objective:**

This review explores the multifunctional role of smart bioactive hydrogels in MI repair, focusing on their stimuli-responsive drug delivery, electroconductive properties, and biosensing potential.

**Methods:**

This narrative review synthesized recent advances in multifunctional smart bioactive hydrogels for MI repair, focusing on systems integrating stimuli-responsive drug delivery, electroconductivity, and real-time biosensing. A comprehensive literature search was conducted in PubMed, Scopus, Web of Science, and Google Scholar for studies published between 2010 and 2025 using relevant keywords. Articles were included if they addressed hydrogel-based platforms featuring at least one of the following: responsive drug release (e.g., pH, temperature, and enzymatic), conductive components (e.g., carbon nanotubes and graphene), or embedded biosensing technologies. Studies limited to conventional hydrogels without multifunctionality were excluded. Relevant data were extracted and thematically categorized by material composition, functional properties, regenerative potential, and translational applicability, with emphasis on preclinical cardiac models. No quantitative synthesis was performed due to heterogeneity across study designs.

**Results:**

Smart bioactive hydrogels have demonstrated significant potential for MI repair by integrating stimuli-responsive drug delivery, electroconductivity, and biosensing within a single therapeutic platform. pH-, ROS-, and enzyme-sensitive systems enable localized, on-demand release of angiogenic factors or cardioprotective drugs, leading to 20–45% infarct size reduction and 1.5–2.3-fold increases in neovascular density in preclinical models. Incorporation of conductive materials such as graphene oxide (GO), polypyrrole, or carbon nanotubes (CNT) has been shown to restore electrical coupling, improve connexin-43 expression, and enhance left ventricular ejection fraction by 8–15%, while narrowing QRS complex duration by ~15 ms in large-animal studies. Emerging biosensing-enabled hydrogels permit real-time monitoring of local biochemical cues, such as pH, oxygen levels, and inflammatory cytokines, maintaining stable signal fidelity for up to 4 weeks without adverse tissue reactions. Advances in 3D/4D bioprinting now allow spatially patterned integration of these functionalities, enabling region-specific therapeutic release and conductivity optimization. Collectively, these multifunctional hydrogels exhibit superior regenerative outcomes compared to conventional scaffolds and hold strong translational promise, although variability in experimental design, lack of standardized endpoints, and limited long-term clinical data remain challenges to widespread adoption.

**Conclusion:**

Smart bioactive hydrogels represent a transformative approach in MI repair by combining structural support with multifunctional properties. Their ability to deliver therapeutics on demand, enhance electroconductivity, and enable real-time biosensing offers new possibilities for precision cardiac medicine.

## Background and significance

Myocardial infarction (MI), commonly referred to as a “heart attack,” arises from a reduction or complete cessation of blood supply to a specific region of the myocardium. This condition may remain asymptomatic and undiagnosed or manifest as a severe, life-threatening event characterized by hemodynamic instability and sudden cardiac death^[1]^. The majority of MIs are attributed to underlying coronary artery disease, which remains the principal cause of mortality in the USA. The obstruction of a coronary artery results in myocardial oxygen deprivation, and prolonged ischemia can culminate in myocardial cell death and necrosis^[1]^.

Annually, over 3 million individuals experience ST-elevation MI (STE-MI), while more than 4 million cases are attributed to STE-MI pathology^[2]^. Although MI is predominantly diagnosed in developed nations, it is also frequently observed in developing countries. A study involving 19 781 patients with coronary artery disease reported an MI prevalence of 23.3%. Although recent decades have seen a decline in STE-MI incidence across Europe and the USA^[1]^, MI remains the leading global cause of death^[1]^. In the USA alone, heart failure affects over 6 million individuals, causes 30 000 deaths annually, and imposes costs approaching $40 billion^[1]^, underscoring the enduring clinical and economic burden of MI^[1]^.

Despite advancements in MI treatment, limitations remain across pharmacotherapy, stem cell therapy, and tissue engineering. Pharmacological agents reduce ischemic events but do not regenerate myocardium and pose bleeding risks. Stem cell therapy struggles with poor retention, immune rejection, and inconsistent outcomes. Tissue engineering faces challenges in vascularization, integration, and scalability^[2,3]^.

Hydrogels are promising biomaterials for myocardial repair due to their biocompatibility, tunable mechanical properties, and capacity to support tissue regeneration^[4]^. These polymeric networks mimic the extracellular matrix, provide structural support, and enable localized delivery of therapeutic agents such as stem cells and growth factors. Advances in biomimetic design, responsive drug release, and enhanced mechanical stability further strengthen their potential in cardiac tissue engineering, making hydrogels a viable strategy for improving MI treatment outcomes^[4]^.

This narrative review aims to critically examine the multifunctional roles of smart bioactive hydrogels in MI therapy, focusing on their capacity for stimuli-responsive drug delivery, electroconductive scaffolding, and real-time biosensing, while highlighting preclinical outcomes and translational potential.

## Methods

**Figure 1.**
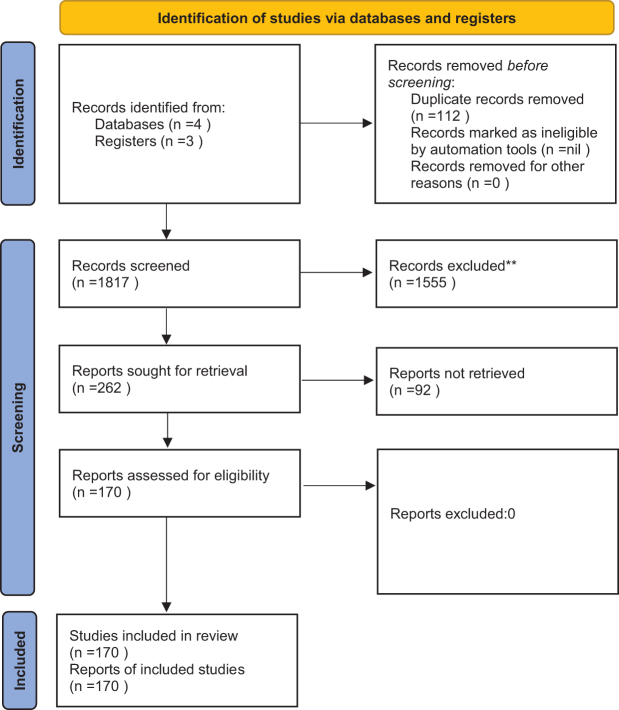
PRISMA flowchart.

This narrative review systematically examined recent advances in smart bioactive hydrogels for MI repair, emphasizing multifunctional platforms that integrate stimuli-responsive drug delivery, electroconductive properties, and real-time biosensing. A comprehensive literature search was conducted in PubMed, Scopus, Web of Science, and Google Scholar for publications dated from 2010 to 2025. Search terms included “smart hydrogel,” “myocardial infarction,” “electroconductive hydrogel,” “stimuli responsive drug delivery,” “3D bioprinting,” “4D bioprinting,” “graphene,” “carbon nanotube,” “MXene,” and “biosensing” (Fig. 1).

**HIGHLIGHTS**

- *Multifunctional hydrogels* offer a promising strategy for myocardial infarction (MI) repair by combining structural support with therapeutic functionality.
- Integration of *stimuli-responsive drug delivery* systems enables on-demand, localized release of growth factors, anti-inflammatory agents, or cardioprotective drugs.
- *Electroconductive hydrogels* enhance electrical signal propagation across damaged myocardium, supporting synchronized cardiac contractility.
- *Embedded biosensing components* allow for real-time, *in situ* monitoring of cardiac biomarkers, pH, and tissue remodeling.
- Advances in material engineering, including *nanomaterial incorporation* and bioinspired designs, improve biocompatibility, mechanical strength, and cell integration.

Inclusion criteria encompassed studies investigating hydrogel systems with at least one of the following functional attributes: responsive drug release (pH, temperature, enzymatic, or reactive oxygen species), incorporation of conductive components (carbon nanotubes, graphene derivatives, or MXene), or integration with biosensing technologies. Studies limited to conventional, nonfunctional hydrogels were excluded. Data extraction focused on hydrogel composition, physicochemical and biological properties, regenerative potential, preclinical cardiac model, and translational applicability. Outcomes of particular interest included infarct size reduction, improvement in left ventricular ejection fraction, vascular density, cardiomyocyte (CM) viability, and integration with host tissue. Due to substantial heterogeneity in study designs, quantitative meta-analysis was not undertaken; instead, a thematic synthesis of preclinical findings was performed. In line with current publishing standards on responsible AI usage and reporting, this study was conducted and reported in compliance with the TITAN 2025 Guidelines on the transparent integration of AI in academic work^[5]^.

### Hydrogels in cardiac tissue engineering

Common natural hydrogels, such as collagen, gelatin, laminin, Matrigel, hyaluronic acid (HA), alginate, and chitosan, closely resemble biological structures, thereby minimizing the likelihood of immune responses following *in vivo* implantation^[4]^. The use of natural and synthetic hydrogels in MI models has been extensively investigated in recent years. Alginate, a widely studied natural hydrogel, has shown mixed results in rodent MI models, with some studies reporting improved cardiac function, increased scar thickness, and enhanced myofibroblast recruitment following intramyocardial injection, while others found no significant functional benefits^[6]^. Collagen-based hydrogels, frequently tested in rodent and swine MI models, have demonstrated the greatest efficacy when administered shortly after ischemia onset^[7]^. Collagen patches combined with adipose-derived stem cells (ADSCs) have significantly improved cardiac function, reduced fibrosis, and promoted angiogenesis and cardiac remodeling in both rat and swine MI models^[7]^ (Table 1).

**Table 1:** Hydrogels in cardiac tissue engineering

Material	Key additives	Animal model	Delivery method	Cardiac function impact	Effect on scar formation	Notable advantages
Alginate	None	Rat MI	Intramyocardial injection	Improved function	Thicker scar, reduced expansion	Encouraged myofibroblast activity
	ASCs	Rat MI	Intramyocardial injection	↑ Ejection fraction	Not specified	Limited added benefit vs. hydrogel alone
	CapGel	Rat MI	Intramyocardial injection	Enhanced LV performance	Not specified	Sustained Ang-(1–7) release, macrophage recruitment
Collagen	None	Mouse MI	Intramyocardial injection	↑ Cardiac output	Scar size ↓ ~40%	Boosted vessel density, reduced apoptosis
	Rat ADSCs	Rat and Pig MI	Epicardial implantation	Supported cell retention	Reduced fibrosis and scar size	Promoted angiogenesis and recovery
Fibrin	Skeletal Myoblasts	Rat I/R	Intramyocardial injection	Not reported	↓ Infarct size	↑ Arteriole density, improved graft survival
	rAAV9-cyclinA2	Rat MI	Intramyocardial injection	Enhanced function	↓ Fibrosis	Stimulated angiogenesis
	BMSCs	Rat MI	Epicardial implantation	Significant improvement	Less fibrosis	↑ Vessel formation, cytokine release
Gelatin	bFGF	Rat MI	Infarct application	Strengthened contractility	↑ Collagen III/I ratio	Stimulated capillary growth
	bFGF	Canine MI	Infarct application	Improved function	Not reported	Enhanced vascularization
	CMs	Rat MI	Intramyocardial injection	Enhanced function	Minimal scar reduction	Encouraged vascular development
PEG	None	Rat MI	Intramyocardial injection	Maintained function	Preserved healthy tissue	Biomimetic, immune-modulating
	VEGF	Rabbit MI	Intramyocardial injection	↑ Heart efficiency	↓ Fibrosis	Stimulated angiogenesis
	None	Pig MI	Epicardial implantation	Strengthened function	↓ Collagen	Promoted vessel formation, cell survival
Hyaluronic acid	None	Rat MI	Intramyocardial injection	↑ Efficiency, reduced remodeling	↓ Fibrosis	Promoted angiogenesis
	MSCs	Mouse MI	Intramyocardial injection	↑ Function and contractility	↓ Infarct size	Supported MSC retention, ↑ vessel density
	IGF-1	Rat MI	Intramyocardial injection	↑ Ejection fraction	↓ Scar formation	Enhanced survival and vessel growth

Collagen, a primary structural protein, contributes significantly to the mechanical strength of tissues. Among the various collagen types present in the human body, type I collagen is the most abundant and widely utilized in tissue engineering due to its excellent biocompatibility. Type I collagen hydrogel can be synthesized by dissolving collagen in a 0.3% glacial acetic acid solution, followed by neutralization to a physiological pH of 7.4^[7]^. However, injecting acidic solutions into infarcted cardiac tissue may induce localized inflammation, while slow gelation time poses additional challenges in cardiac tissue engineering^[7]^ (Table 1).

Fibrin, another natural hydrogel, is formed through the interaction of fibrinogen and thrombin during the coagulation process, catalyzed by calcium^[8]^. As a nontoxic and biodegradable biomaterial, fibrin holds significant potential for tissue engineering applications. Birla *et al* reported the implantation of hollow fibrin gel tubes filled with neonatal CMs into adult rat femoral arteries, highlighting its feasibility for myocardial repair^[8]^. Additionally, Huang *et al* implanted rat CMs in fibrin gel and observed sustained contractility and normal pacing ability for up to 2 months^[9]^. Black *et al* further explored fibrin/CM constructs under conditions of radial contraction, demonstrating their potential utility in myocardial tissue engineering^[10]^ (Table 1).

### Synthetic polymeric hydrogels

Synthetic polymers, including poly(ethylene glycol) (PEG), polylactide (PLA), polylactide-glycolic acid (PLGA), polycaprolactone (PCL), polyacrylamide (PAA), and polyurethane (PU), are widely utilized in cardiac tissue engineering due to their customizable physical and chemical properties, such as modulus, water affinity, and degradation rate, making them well-suited for myocardial repair^[11]^. However, a key concern with synthetic polymers is their potential cytotoxicity. Among the commonly used polymers in drug delivery, PEG, PLA, and PLGA are extensively studied, while PAA and PU have demonstrated biocompatibility in both *in vitro* and *in vivo* studies^[12,13]^.

PEG, a water-soluble polymer synthesized via ring-opening polymerization of ethylene oxide, is widely employed in drug delivery and tissue engineering due to its biocompatibility. Photopolymerization of diacrylate-modified PEG in the presence of UV light leads to the formation of PEG hydrogels, which serve as supportive matrices in various tissue engineering applications, including the liver, pancreas, bladder, skin, and cartilage^[13]^. However, its low protein affinity limits cell adhesion, necessitating modifications such as protein or peptide conjugation and the incorporation of growth factors. PEG hydrogels have also been used to study three-dimensional (3D) CM–matrix interactions^[13]^.

Poly(2-hydroxyethyl methacrylate) (PHEMA) is another hydrophilic polymer with pendant hydroxyl groups that has been explored for cardiac applications^[14]^. Walker *et al* investigated its use in the canine epicardium by embedding a poly(ethylene terephthalate) (PET) mesh-reinforced PHEMA gel, highlighting its potential for myocardial repair^[15]^. PAA gels, owing to their amide structure-mimicking properties, hold promise for cardiac tissue engineering. Similar to PEG, PAA hydrogels benefit from crosslinking techniques and exhibit a thermo-responsive sol–gel transition, allowing them to remain in liquid form at lower temperatures and transition into a soft gel at physiological temperatures. This unique property makes PAA hydrogels ideal for injectable applications, enabling minimally invasive delivery^[13]^. Poly(*N*-isopropylacrylamide) (PNIPAM) is a widely studied thermosensitive polymer with a lower critical solution temperature of 32°C, remaining in a liquid state at room temperature and forming a gel at physiological temperatures^[16]^.

Injectable hydrogels are ideal for deep or difficult-to-access infarct regions, and patches offer superior spatial control over cell and drug distribution^[13,17]^. However, patch application requires open-heart surgery, making it more invasive than catheter-delivered injectable systems^[4]^. Injectable hydrogels must rapidly transition from liquid to gel at the target site to prevent washout in the highly vascularized cardiac environment, whereas hydrogel patches are surgically placed onto the myocardium, ensuring immediate mechanical support. Both approaches have their advantages, and the choice between them depends on factors such as infarct location, mechanical stability needs, and clinical feasibility^[17]^. While injectable hydrogels offer minimally invasive delivery and *in situ* gelation, allowing them to conform to irregularly shaped myocardial defects, patch-based hydrogels provide a preformed, structurally stable scaffold that can deliver cells or bioactive agents in a controlled manner(Table 2).

**Table 2:** Injectable hydrogels vs patch-based hydrogels

Feature	Injectable hydrogels	Patch-based hydrogels
Delivery method	Injected via catheter or syringe directly into the myocardium.	Applied as a preformed scaffold on the epicardial surface of the heart, usually through surgical implantation.
Invasiveness	Minimally invasive; can be delivered percutaneously without the need for open-heart surgery.	Requires open-chest surgery for implantation, increasing surgical risks.
Gelation mechanism	Requires *in situ* gelation; controlled by stimuli like temperature, pH, or light. Needs precise control to avoid premature gelation in the catheter.	Prefabricated with defined mechanical properties, it does not rely on *in situ* gelation.
Retention and stability	Prone to washout due to blood flow, especially if gelation is slow. Stability depends on polymer composition and crosslinking.	Remains fixed on the heart surface, ensuring better stability and retention of therapeutic agents.
Mechanical strength	Generally weaker due to a fluid-like nature before gelation. Can be adjusted by modifying polymer concentration, but still less robust than patches.	Provides strong mechanical support to the infarcted area, helping counteract myocardial wall thinning and excessive strain.
Target area	Can penetrate deep into the myocardium, allowing localized treatment of infarcted regions.	Primarily affects the outer (epicardial) layer of the heart, limiting direct impact on deeper myocardial tissue.
Therapeutic agent delivery	Can encapsulate stem cells, growth factors, or drugs for controlled release, but cells may disperse or wash away before engraftment.	Offers better containment of bioactive agents, preventing early washout and ensuring sustained delivery over time.
Cellular integration and engraftment	Challenges with stem cell retention and differentiation due to the fluid nature; rapid gelation may trap cells unevenly.	Provides a structured environment for cells, enhancing engraftment, differentiation, and tissue regeneration.
Vascularization potential	Can promote vascularization if loaded with angiogenic factors, but may not provide sufficient structural support for new vessel growth.	Supports neovascularization more effectively by acting as a scaffold for endothelial cell infiltration and vessel formation.
Degradation rate and biocompatibility	Highly tunable degradation, depending on polymer composition. Faster degradation may lead to premature material loss.	It can be designed for long-term presence, offering extended structural support while gradually degrading.
Clinical translation and feasibility	Several formulations have entered preclinical and early clinical trials, mainly for minimally invasive therapies.	Advancing toward clinical use, but surgical implantation remains a barrier to widespread adoption.
Cost and scalability	Generally, more scalable and cost-effective due to its injectable nature and off-the-shelf potential.	More expensive due to fabrication complexity and surgical requirements.

### Functional enhancements for myocardial repair

Hydrogels have emerged as versatile platforms for myocardial repair due to their ability to deliver bioactive molecules, stem cells, and extracellular vesicles (EVs) in a controlled manner, thereby promoting angiogenesis, CM survival, and immunomodulation. Angiogenesis, essential for restoring blood supply to ischemic myocardium, is tightly regulated by growth factors such as VEGF, PDGF-BB, bFGF, HGF, IGF, and TGF-β^[18]^. The therapeutic efficacy of these factors depends on precise release kinetics, as insufficient or excessive exposure can result in unstable or aberrant vessel formation. Hydrogel scaffolds have been engineered to modulate growth factor delivery through strategies, such as covalent crosslinking with heparin or fibrin, incorporation of proteolytically degradable microspheres, functionalization with bioactive peptides (e.g., RGD, REDV), and layer-by-layer polymer assembly^[19,20]^. These approaches enhance endothelial cell attachment, migration, and proliferation, facilitating the formation of stable vascular networks while mitigating burst release. Notably, multicomponent delivery systems – such as heparin-immobilized scaffolds or fibronectin-functionalized hydrogels – demonstrate superior angiogenic outcomes compared to single-factor delivery, emphasizing the importance of recapitulating the complex signaling environment of the extracellular matrix.

Beyond growth factor delivery, hydrogels serve as carriers for stem cells and EVs, offering additional regenerative and immunomodulatory benefits. Stem cell-derived EVs exhibit higher bioactive cargo content than somatic cell EVs, and their composition can be influenced by tissue origin, donor age, sex, and environmental conditions^[21]^. To circumvent ethical and scalability limitations of cardiac stem cells, alternative sources such as mesenchymal stem cells, endothelial progenitor cells (EPCs), and induced pluripotent stem cell (iPSC)-derived CMs have been employed. Chen *et al* demonstrated that EPC-derived EVs encapsulated in a shear-thinning HA hydrogel reduced infarct size and enhanced angiogenesis in a rat MI model, as indicated by increased vWF, α-SMA, and CD11b-positive cells in the peri-infarct region^[22,23]^. Similarly, self-assembling peptide amphiphile hydrogels incorporating cardiac protective peptides and MSC-derived EVs promoted vascularization, attenuated fibrosis, suppressed apoptosis, and modulated TGF-β1 signaling^[24]^.

iPSC-derived CM EVs delivered via collagen Gelfoam hydrogel patches further illustrate the critical interplay between EV type and hydrogel properties. Liu *et al* reported sustained EV release, reduced infarct size, mitigated pathological hypertrophy, and improved ejection fraction in athymic rats, while CM-derived EV patches outperformed iPSC-EV patches in reducing Caspase activity and apoptosis^[25]^. Immunomodulatory strategies using dendritic cell-derived EVs (DEXs) encapsulated in alginate hydrogels have also been shown to prolong EV retention, reduce M1 macrophage infiltration, increase CD206+ macrophages and regulatory T cells, and enhance vascularization, collectively contributing to improved myocardial repair^[26]^.

Critically, these studies underscore the importance of hydrogel design, including composition, degradation kinetics, and mechanical properties, in determining therapeutic outcomes. While promising, challenges remain in optimizing spatiotemporal growth factor release, EV bioactivity, and immunomodulatory effects to fully replicate the complex myocardial microenvironment (Table 3).

**Table 3:** Various studies supporting the use of hydrogels in cardiac regeneration

Study	EV source	Hydrogel type	Key findings
Chen *et al*	Rat endothelial progenitor cells (EPCs)	Shear-thinning hyaluronic acid (HA)	Reduced infarct size, increased angiogenesis (vWF, α-SMA, CD11b+ cells), improved ventricular remodeling, and cardiac function.
Han *et al*	Human umbilical cord MSCs	Peptide amphiphile (PA) hydrogel with cardiac protective peptides (GHRPS)	Reduced fibrosis, downregulated TGF-β1, decreased apoptotic cardiomyocytes, increased CD31+ vascular cells, and reduced inflammation (CD68+ cells).
Liu *et al*	iPSC-derived cardiomyocytes	Collagen gelfoam mesh	Sustained EV release, reduced infarct size and pathological hypertrophy, improved ejection fraction.
Zhang *et al*	Dendritic cells (DEXs)	Alginate hydrogel (sodium and calcium alginate)	Prolonged EV retention, reduced iNOS+ M1 macrophages, increased CD206+ macrophages and Foxp3+ Treg cells, and enhanced vascular density (CD31+ cells).

Replicating the structural complexity of native cardiac tissue is crucial for engineering functional myocardial constructs. Scaffold porosity and microarchitecture significantly influence tissue formation by affecting matrix stiffness, extracellular matrix (ECM) secretion, and cellular behavior, including survival, proliferation, and migration^[27]^. Traditional scaffold fabrication techniques, such as solvent casting, freeze-drying, and gas foaming, allow for control over pore size and porosity but lack precision in creating complex geometries or guiding cell distribution necessary for biomimetic cardiac tissue engineering^[27]^. Advancements in biofabrication, including microfabrication tools, fiber-based technologies, and 3D bioprinting, have overcome these limitations by enabling precise control over scaffold mechanical properties, pore interconnectivity, and cellular organization^[28]^. These technologies have been widely applied in cardiac tissue engineering due to their ability to generate highly structured, cell-laden constructs. Among these, photopatterning and micromolding have emerged as leading microfabrication approaches for hydrogel-based cardiac scaffolds, allowing fine-tuned cell–material interactions and improved myocardial tissue integration^[27,28]^.

Photopatterning, or photolithography, employs light exposure to create micropatterned hydrogels by selectively crosslinking regions of a photocrosslinkable polymer, such as gelatin methacryloyl (GelMA) or methacrylated HA. This method provides spatial control over the cellular microenvironment and enables the incorporation of multiple cell types within a single construct^[29]^. However, challenges such as optimal UV exposure time and resolution limitations at higher wavelengths remain. Studies have shown that photopatterned GelMA hydrogels loaded with endothelial cells and cardiac progenitors exhibit high cell viability, with microconstruct width influencing cellular alignment and elongation – critical factors for CM organization and function^[29]^.

Micromolding, another promising technique, utilizes preformed molds made from materials like poly(methyl methacrylate) (PMMA) or polydimethylsiloxane (PDMS) to create highly defined hydrogel structures^[30]^. This cost-effective and scalable approach allows for the fabrication of complex cardiac constructs with high resolution. Additionally, layer-by-layer assembly, an extension of micromolding, enables the sequential deposition of oppositely charged polymers, facilitating the controlled release of bioactive molecules such as growth factors or small molecules essential for cardiac regeneration^[31]^. These emerging biofabrication strategies offer precise control over scaffold architecture, enhancing cell organization, function, and ultimately, the development of engineered myocardial tissue^[23,30]^ (summarized in Table 4).

**Table 4:** Hydrogels for cardiomyocyte regeneration

Hydrogel	Approach	Results
GelMA	Nanofunctionalization with CNTs, GO, and rGO	Enhanced electrophysiological properties, electrical conductivity, mechanical stiffness, and CM maturation
	3D bioprinting + Fibronectin	Improved CM survival and spreading
	3D bioprinting + GNRs	Facilitated CM spreading and electrical signal propagation
	Nanofunctionalization with GNW	Enhanced CM contractile behavior and maturation
Chitosan	Nanofunctionalization with AuNPs and GO	Controlled degradation, CM maturation, increased conductivity, improved heartbeat, and electrical activity *in vivo*
Collagen	Nanofunctionalization with AuNPs	Increased CM maturation, infarcted myocardium recovery, and reduced scar size
	Nanofunctionalization with CNTs	Improved cardiac function and contraction
Alginate	Injection	Promising potential for cardiac regeneration
	Nanofunctionalization with peptides	Enhanced CM attachment, maturation, and alignment

### Electroconductive hydrogels for improved electrical integration

Electroconductive hydrogels (ECHs) have emerged as a transformative strategy in cardiac tissue engineering by enhancing electrical integration and promoting myocardial remodeling^[32]^. These hydrogels combine a biocompatible matrix with conductive components to mimic the electrophysiological properties of native myocardium, supporting CM alignment, differentiation, and functional coupling. By restoring electrical signaling, ECHs facilitate synchronized contraction, gap junction formation, and extracellular matrix (ECM) remodeling, which are critical for reducing fibrosis and supporting cardiac regeneration^[32,33]^.

ECHs can be broadly categorized into ionic, metallic, and polymer-based systems. Ionic conductive hydrogels, containing positively and negatively charged groups within a porous 3D structure, support ion transport, a key determinant of electrophysiological function. However, their applications are often limited by suboptimal biocompatibility and insufficient self-healing properties^[33]^. Metallic-based hydrogels offer superior electrical conductivity and mechanical stability, yet crosslinking between metal particles and polymer chains can compromise their performance in biological environments^[34]^. To overcome these limitations, conductive polymers such as polypyrrole (PPy), polyaniline (PANI), and poly-(3,4-ethylene dioxythiophene) (PEDOT) have been incorporated into hydrogels, providing a balance of elasticity, conductivity, and biocompatibility^[35]^. The hydrophilic polymer network ensures structural stability, while the conductive components facilitate charge transport and dynamic swelling behavior, enhancing responsiveness to the cardiac microenvironment^[33,34]^.

Fabrication strategies for ECHs include integrating conductive polymers, introducing ionic carriers, and embedding conductive nanomaterials such as GO, CNTs, and gold nanowires (GNWs). Conductive polymers offer efficient charge transport via conjugated π-electron structures, while ionic carriers optimize electrophysiological responses by facilitating ion flow. Nanomaterial incorporation further enhances conductivity, mechanical resilience, and CM maturation, although potential cytotoxicity and long-term biocompatibility remain concerns^[35,36]^.

Beyond electrical signaling, ECHs provide platforms for controlled, electrically-responsive drug delivery. Mechanisms such as electrically-triggered diffusion, redox-switching, and electro-responsive erosion enable precise, on-demand release of bioactive molecules, antifibrotic agents, or pro-survival factors. These properties allow ECHs to function not only as structural scaffolds but also as dynamic therapeutic reservoirs, integrating tissue engineering and regenerative pharmacology^[33]^.

### Carbon-based and MXene ECHs for cardiac repair

Carbon-based ECHs are increasingly investigated in cardiac tissue engineering for their ability to replicate the electrical conductivity of native myocardium, promote CM maturation, and facilitate synchronized contraction^[37]^. Among these materials, graphene and its derivatives have shown particular promise. Graphene, a two-dimensional (2D) hexagonal carbon lattice, offers exceptional electrical conductivity, mechanical strength, and thermal stability, making it well-suited for myocardial tissue engineering. Incorporation of GO or reduced graphene oxide (rGO) into hydrogels enables fine-tuning of mechanical stiffness and bioactivity, supporting CM attachment, electrical signal propagation, and integration with host tissue^[38]^. However, the hydrophobicity and aggregation tendency of graphene present challenges, requiring advanced dispersion strategies such as wet spinning or *in situ* redox processes to maintain uniform conductivity and hydrogel stability. Biocompatibility assessment remains a critical step for clinical translation^[37]^.

CNTs, characterized by their high aspect ratio and exceptional electrical and mechanical properties, have also been incorporated into ECHs to enhance CM contractility and synchronous beating^[38]^. CNT-based hydrogels facilitate electrical coupling and mechanical reinforcement, which are essential for functional cardiac patches and engineered heart tissues. Nonetheless, CNTs’ strong van der Waals forces, hydrophobicity, and aggregation tendency complicate homogeneous hydrogel formulation. Surface functionalization and dispersing agents, including microgel particles, have been employed to improve integration. Despite these strategies, potential cytotoxicity and inflammatory responses require thorough biocompatibility validation prior to clinical application^[38,39]^.

MXenes, a class of 2D transition metal carbides, nitrides, or carbonitrides, have emerged as promising conductive nanomaterials for cardiac ECHs due to their high electrical conductivity, hydrophilicity, and inherent biocompatibility^[40]^. MXene-integrated hydrogels support CM adhesion, proliferation, and electrical signaling, making them attractive for injectable cardiac patches aimed at restoring conductivity in infarcted myocardium^[41]^. However, their low dispersibility in aqueous environments necessitates surface modifications and self-assembled hydrogel platforms to enhance stability and uniform performance in cardiac applications^[40]^.

#### Metal nanoparticles

Metal nanoparticles, including gold, silver, platinum, and iron oxide, have also been widely investigated for their role in enhancing the properties of cardiac ECHs. These nanoparticles offer unique electrical, optical, and bioactive properties that contribute to improved CM function and myocardial regeneration. Gold nanoparticles (AuNPs), for instance, have been integrated into ECHs to enhance electrical conductivity and promote CM maturation, leading to improved contractility and reduced fibrosis in engineered cardiac tissues^[42]^. Similarly, silver and platinum nanoparticles have been employed to develop ECHs with antimicrobial properties, reducing the risk of infection in implantable cardiac patches. However, challenges such as high production costs, cytotoxicity, and potential off-target effects must be addressed to optimize the clinical translation of metal nanoparticle-based ECHs for cardiac applications^[43,44]^.

### Intelligent hydrogels and cardiac tissue engineering

#### Stimuli-responsive hydrogels for cardiac regeneration

Hydrogels, water-rich polymeric biomaterials resembling the cardiac extracellular matrix (ECM), can undergo structural rearrangements in response to environmental cues, enabling their design as stimuli-responsive or “intelligent” hydrogels. These systems are engineered to undergo controlled transformations *in vivo*, responding to intrinsic or extrinsic stimuli such as temperature, pH, ions, hypoxia, and reactive oxygen species (ROS). By adapting to local conditions, intelligent hydrogels offer multifunctional capabilities in regenerative cardiology, including localized drug delivery, structural support for injured myocardium, angiogenic factor release, and enhancement of electrical conductivity.

*Temperature-responsive hydrogels* transition reversibly between liquid and gel states based on thermal cues, exploiting polymers such as PNIPAAm, PLGA-PEG-PLGA, Pluronics® F-127, PVCL, and PDMAEMA^[45,46]^. These hydrogels can be delivered minimally invasively in liquid form and solidify upon reaching physiological temperature, providing localized mechanical support and enabling sustained delivery of therapeutic agents. Preclinical studies have demonstrated improved cardiac regeneration, enhanced cell retention, and reduced postinfarction remodeling when temperature-responsive hydrogels are combined with pro-angiogenic drugs or stem cell therapies^[23,40,47]^. Despite these advantages, challenges remain in fine-tuning gelation kinetics, mechanical strength, and long-term biocompatibility.

*pH-responsive hydrogels* exploit acidic or basic microenvironments to trigger hydrogel swelling or dissolution. In infarcted myocardium, where the local pH is lower than in healthy tissue, these hydrogels enable targeted delivery of growth factors and regenerative agents^[48,49]^. Composite pH- and temperature-responsive systems have demonstrated self-healing properties, enhanced angiogenesis, CM proliferation, macrophage modulation, and reduced fibrosis in preclinical MI models^[50,51]^. Nonetheless, precise control over drug release kinetics and hydrogel stability under fluctuating physiological pH remains a critical barrier for clinical translation.

*Ion-sensitive hydrogels* respond to changes in ionic strength or specific metal ions, undergoing phase transitions that influence swelling, shape, and conductivity^[52]^. These properties facilitate CM assembly into functional syncytia, improving electrical coupling and contractility. Their ionic nature also allows controlled adsorption and release of charged biomolecules, enhancing drug delivery precision^[53,54]^. However, ensuring uniform ion responsiveness and mechanical stability *in vivo* is challenging, particularly within the dynamic ionic environment of the infarcted heart.

*Hypoxia-responsive hydrogels* are designed to sense oxygen-deficient regions and release therapeutic agents selectively to ischemic tissue^[55]^. By delivering angiogenic and cardioprotective factors while sustaining local oxygen levels, these hydrogels promote vascularization, reduce fibrosis, and support CM survival^[56]^. Key limitations include the complexity of designing materials that respond precisely to variable oxygen gradients and maintaining hydrogel stability under prolonged hypoxic conditions.

*ROS-responsive hydrogels* target oxidative stress, a major contributor to post-MI cardiac dysfunction and fibrosis^[57]^. Redox-sensitive components trigger the release of antioxidants, anti-inflammatory agents, or regenerative biomolecules when ROS levels rise, thereby mitigating oxidative damage, limiting CM apoptosis, and enhancing tissue repair^[33,58]^. ROS-responsive hydrogels also provide protective microenvironments for therapeutic cells, improving survival and integration. Challenges include ensuring selective responsiveness to pathological ROS levels without interfering with physiological signaling and maintaining structural integrity during repeated oxidative cycles.

### Advanced technologies in hydrogels for cardiac applications

#### 3D and 4D bioprinting

3D bioprinting is an emerging technology in tissue engineering and regenerative medicine that enables the fabrication of complex biomimetic structures replicating the architecture and function of native tissues, including the heart. This process involves the precise deposition of bioinks containing various cell types, such as CM, endothelial cells, fibroblasts, mesenchymal stem cells, and smooth muscle cells, within a hydrogel scaffold^[59]^. Bioinks, composed of biomaterials capable of transitioning from a liquid to a solid state under controlled conditions, provide structural support while promoting cell survival, organization, and function. Beyond structural support, 3D-printed hydrogels serve as drug delivery systems, enabling controlled and targeted therapeutic interventions for conditions such as MI. They can also be used to evaluate the safety and efficacy of novel drug delivery strategies prior to clinical application^[13]^.

Hydrogels used in bioprinting must exhibit tunable viscosity, biocompatibility, mechanical strength, biodegradability, and sufficient porosity to allow proper nutrient diffusion^[13]^. Common biomaterials include alginate, chitosan, agarose, collagen, HA, and decellularized extracellular matrix, which collectively provide a microenvironment that mimics the heart’s natural extracellular matrix and promotes essential cellular interactions^[60]^.

Recent studies highlight the potential of hydrogel-based 3D bioprinting for cardiac repair. For example, Maiullari and colleagues developed a polyethylene glycol-based hydrogel embedded with induced pluripotent stem cell-derived CMs and human umbilical vein endothelial cells. When transplanted into immunodeficient mice, the constructs matured *in vivo* and promoted vascularization, demonstrating potential for ischemic heart disease therapy^[61]^. Similarly, hydrogel patches composed of HA and gelatin combined with endothelial and mesenchymal stem cells improved cardiac function and reduced adverse remodeling in a mouse model of MI over 8 weeks, supporting the regenerative promise of these materials^[62]^.

Four-dimensional (4D) bioprinting advances these approaches by incorporating the element of time, allowing printed constructs to undergo controlled transformations after printing. This enables dynamic changes in cell organization and tissue structure, more closely mimicking the adaptability of natural cardiac tissue. 4D-printed constructs can be shaped into specific geometries, such as tubular structures resembling blood vessels, or guided to self-assemble through external stimuli. Studies demonstrate that cardiac myocytes exposed to chemical stimuli form gap junctions, enhancing electrical conductivity and synchronized contraction – key features for functional cardiac grafts^[62,63]^. By integrating 4D bioprinting with biomaterial engineering, hydrogel constructs achieve precise architecture, improved vascularization, and enhanced tissue integration, thereby paving the way for advanced myocardial repair strategies^[64]^.

Artificial intelligence is increasingly integrated with hydrogel-based therapies to optimize design, fabrication, and application, improving precision, personalization, and therapeutic efficiency. A notable example is the development of thermoresponsive, injectable hydrogels that adapt to cardiac tissue properties upon administration. A poly(*N*-isopropylacrylamide-co-*N*-vinylpyrrolidone-co-MAPLA) hydrogel with temperature-dependent stiffness was injected into infarcted porcine hearts using a robotic system. AI-guided, temperature-controlled delivery ensured accurate placement and penetration depth, avoiding complications such as occlusion^[65,66]^. The integration of AI with hydrogel technology holds promise for real-time monitoring, predictive modeling, and personalized regenerative therapies, ultimately accelerating the development of smart biomaterials capable of responding to dynamic cardiac environments^[56,64]^.

### Integration of electroconductivity, drug delivery, and bioprinting for advanced cardiac therapies

ECHs have evolved from niche biomaterials into multifunctional therapeutic platforms, offering a convergence of properties that extend well beyond isolated applications in cardiac tissue engineering. Their intrinsic ability to restore myocardial electrical continuity – by facilitating action potential propagation, synchronized cardiomyocyte contraction, and enhanced gap junction formation – establishes a foundation for functional cardiac recovery^[32–34]^. Importantly, these electroactive matrices are increasingly being engineered to couple electrical stimulation with localized, controllable drug delivery. Through mechanisms such as electrically triggered diffusion, redox-mediated conformational switching, and electro-responsive erosion, ECHs can release regenerative growth factors, antifibrotic compounds, or pro-survival molecules in a spatially and temporally precise manner, thereby integrating electrophysiological restoration with targeted biochemical modulation^[33]^.

Parallel advances in 3D and 4D bioprinting have expanded the potential of ECH-based technologies by enabling the fabrication of complex, biomimetic constructs that faithfully replicate myocardial architecture. Bioprinted hydrogels incorporating CMs, endothelial cells, and stem cell populations can be engineered with conductive polymers, carbon nanostructures, or metallic nanowires to achieve concurrent electroconductivity and drug-loading capacity^[59–63]^. The advent of 4D printing adds a further dimension of functionality, allowing constructs to undergo programmed transformations or modulate therapeutic release in response to physiological stimuli, thus approximating the dynamic adaptability of native cardiac tissue^[63]^.

The deliberate integration of electroconductivity, controlled pharmacological delivery, and advanced bioprinting strategies establishes a translationally relevant paradigm for next-generation cardiac therapies. Rather than functioning as discrete technologies, these modalities converge to yield bioengineered constructs that are electrically active, biologically instructive, and pharmacologically responsive. Such systems have the potential to not only restore electrophysiological integrity but also deliver therapeutics in a highly controlled and adaptive fashion, ultimately reducing fibrosis, enhancing myocardial regeneration, and improving long-term functional outcomes.

### Limitation

The clinical translation of hydrogel-based 3D/4D bioprinting and AI-assisted delivery systems for cardiac regeneration, including MI therapy, is hindered by several interrelated limitations. From a materials standpoint, many hydrogels lack the mechanical resilience and elasticity needed to withstand repetitive myocardial contractions, resulting in premature degradation or displacement, while their inherently poor electrical conductivity limits synchronized contraction unless conductive additives are incorporated, sometimes at the expense of biocompatibility^[46]^. Mechanical weaknesses are compounded by challenges in tuning degradation rates to match tissue regeneration, as excessive breakdown leads to transient effects, whereas prolonged persistence can interfere with remodeling. In drug delivery applications, growth factor denaturation during gelation and uncontrolled burst release due to high porosity reduce therapeutic efficacy, while inadequate retention in highly vascularized myocardium further diminishes outcomes^[23]^. Biologically, the generation of mature, patient-specific CMs at clinically relevant scales remains limited, and hydrogel-delivered stem cells often exhibit low survival and differentiation rates. Vascularization within engineered constructs is still suboptimal, with long-term patency and integration *in vivo* posing unresolved challenges. Immune responses to certain synthetic or modified hydrogels may induce inflammation or fibrosis, undermining biocompatibility^[33]^. Technically, although high-resolution printing is essential for microvascular patterning, it prolongs fabrication times and may compromise cell viability, while controlling postprinting shape transformations in 4D constructs under complex physiological conditions remains imperfect^[42]^. AI integration adds further barriers, including dependence on large, high-quality datasets that are scarce in personalized cardiac regeneration, the high cost and limited accessibility of robotic delivery platforms, and complex regulatory approval pathways for hybrid biologic device systems^[12,47,65]^. Translation to clinical practice is additionally constrained by complex manufacturing processes, storage and scalability limitations, limited large-animal and human trial data, and ethical concerns surrounding stem cell sourcing, AI-driven decision-making, and patient data privacy^[65]^. Overcoming these obstacles will require improvements in mechanical stability, controlled release kinetics, immune compatibility, and manufacturing scalability, alongside rigorous preclinical and clinical validation to facilitate safe and effective adoption in cardiac repair.

### Critical appraisal and study quality considerations

While hydrogel-based strategies for cardiac repair, including electroconductive, carbon- and MXene-enhanced, stimuli-responsive, and 3D/4D-bioprinted systems, show considerable promise, the quality of the underlying studies varies widely. Most evidence derives from preclinical *in vitro* or small-animal models, often with limited sample sizes and short follow-up periods, which may not fully capture long-term safety, efficacy, or functional integration in human myocardium. Variations in hydrogel composition, fabrication methods, and cellular components further complicate comparisons across studies. Few investigations rigorously address reproducibility, blinding, or standardized outcome measures, highlighting potential biases. Consequently, while the findings suggest significant therapeutic potential, cautious interpretation is warranted, and future work should emphasize robust study design, reproducibility, and translational relevance to strengthen confidence in clinical applicability.

## Conclusion

This review highlights recent advancements in cardiac tissue engineering with hydrogels, emphasizing their potential in heart regeneration following MI. Hydrogels, with their bioactivity, biocompatibility, and biodegradability, are ideal for cardiac repair, offering structural support and controlled drug delivery. Their strong binding affinity, swelling capacity, and cross-linked spatial structure enhance agent delivery, prolong *in vivo* stability, and improve conductivity for cardiac contraction. Injectable hydrogels enable localized therapeutic delivery, whereas hydrogel-based patches reduce MI-induced stress and provide mechanical reinforcement. The integration of intelligent hydrogels and 3D/4D printing further expands their applications, making them promising candidates for future cardiovascular treatments. However, limited clinical translation necessitates further research to optimize their therapeutic efficacy and commercial viability.

## Data Availability

Not applicable.

## Transparency statement

This document was prepared with a commitment to transparency, accuracy, and integrity in research. All data, methodologies, and analyses included in this study have been meticulously reviewed and sourced from credible scientific literature. Any potential conflicts of interest have been disclosed, and all efforts have been made to ensure that the findings presented are unbiased and based on robust evidence. The authors have adhered to ethical guidelines for research and publication, including maintaining objectivity and providing a clear and honest account of the research conducted. All sources of funding, if applicable, have been disclosed, and there has been no undue influence from sponsors or external parties in the preparation of this document. We encourage peer review and welcome constructive feedback to further refine and enhance the quality and reliability of this work.
